# Three-Dimensional Identification of Microorganisms Using a Digital Holographic Microscope

**DOI:** 10.1155/2013/162105

**Published:** 2013-03-31

**Authors:** Ning Wu, Xiang Wu, Tiancai Liang

**Affiliations:** ^1^Shenzhen Key Lab of Wind Power and Smart Grid, Harbin Institute of Technology Shenzhen Graduate School, Shenzhen 518055, China; ^2^School of Mechanical and Electrical Engineering, Harbin Institute of Technology, 92 West Dazhi Street, Nan Gang District, Harbin 150001, China; ^3^GRG Banking Equipment Co., Ltd., 9 Kelin Road, Science Town, Guangzhou 510663, China

## Abstract

This paper reports a method for three-dimensional (3D) analysis of shift-invariant pattern recognition and applies to holographic images digitally reconstructed from holographic microscopes. It is shown that the sequential application of a 2D filter to the plane-by-plane reconstruction of an optical field is exactly equivalent to the application of a more general filter with a 3D impulse response. We show that any 3D filters with arbitrary impulse response can be implemented in this way. This type of processing is applied to the two-class problem of distinguishing different types of bacteria. It is shown that the proposed technique can be easily implemented using a modified microscope to develop a powerful and cost-effective system with great potential for biological screening.

## 1. Introduction

In the past, high-resolution imaging of three-dimensional (3D) objects, or matter suspended in a volume of fluid, has mainly been accomplished using confocal microscopes [1]. In recent years, however, attention has returned to wide-field optical microscopy using coherent illumination and holographic recording techniques that exploit advances in digital imaging and image processing to compute 3D images. In contrast, with confocal imaging, coherent microscopy provides 3D information from a single recording that can be processed to obtain imaging modes analogous to dark field, phase or interference contrast as required [2–7]. In comparison with incoherent microscopes, a coherent instrument provides an image that can be focused at a later stage and can be considered as a microscope with an extended depth of field. For screening purposes, the increased depth of field is significant, particularly at high magnifications and high numerical aperture. For example a conventional, high magnification microscope has a depth of field of only a few microns whereas a comparable coherent instrument can have a depth of field of a few millimetres or so. This means that around 1000 times the volume of fluid can be screened from the information contained in a single digital recording [8].

The potential of coherent microscopes for automated biological screening is clearly dependent on the development of robust image or pattern recognition algorithms [9]. In essence, the application of pattern recognition techniques to coherent images is similar to that applied to their incoherent counterpart. The task can be defined as that of highlighting objects of interest (e.g., harmful bacteria) from other clutter (e.g., cell tissue and benign bacteria). This process should be accomplished regardless of position and orientation of the objects of interest within the image. It can be accomplished using variations on correlation processing. Linear correlation processing has been criticized in the past for its lack of rotation invariance and its inability to generalize in the manner of neural network classifiers; however, a cascade of correlators, separated by nonlinear (decision) layers, has considerably enhanced performance [5, 10]. Furthermore, we have shown that this is the architecture a neural network classifier assumes if it is trained to provide a shift-invariant output [11, 12]. 

The application of linear correlation processing to the complex images recorded by a digital phase shifting interferometer has recently been demonstrated by Javidi and Tajahuerce [13]. Pattern recognition techniques implemented using a holographic microscope for the detection of microscale objects has also been considered by Dubois et al. [5, 14] In these works, the 3D sample field was reconstructed plane by plane and image classification was performed by the application of a 2D correlation filter to each of the reconstructed planes. It is noted, however, that although 2D correlation can be applied independently to different image planes it does not take into account the true nature of 3D optical fields, nor that the information in any two planes of these fields is, in fact, highly correlated [15].

In this paper, we considered, from first principles, 3D shift-invariant pattern recognition applied to optical fields reconstructed from digital holographic recordings. It will be shown that the sequential application of a 2D filter to plane-by-plane reconstructions is exactly equivalent to the application of a 3D filter to the full 3D reconstruction of the optical field. However, a linear filter designed based on the plane of focus will not necessarily work for planes out of focus, and therefore a 3D nonlinear filtering scheme is introduced into the optical propagation field. The 3D nonlinear filter is a system implemented with a general impulse response and followed by a nonlinear threshold. We will prove with experiment that a 3D nonlinear filtering structure can significantly improve the classification performance in 3D pattern recognition. In the experiment, we will apply the 3D nonlinear filter to 3D images of two types of bacteria recorded from a holographic microscope, and the enhanced classification performance will be shown.

## 2. Theory

Firstly, we define the 3D cross-correlation of complex functions *u*(**r**) and *h*(**r**) as

(1) $$\begin{array}{c} R\left(r\right)=\int_{-\infty }^{+\infty }u\left(x\right)h\left(x-r\right)dx, \end{array}$$

where **r** is a position vector and *d* **x** conventionally denotes the scalar quantity (*dx*, *dy*, *dz*). Assume that *H*(**k**) and *U*(**k**) are the Fourier transforms of *h*(**r**) and *u*(**r**), respectively; according to the convolution theorem, *R*(**r**) can also be written:

(2) $$\begin{array}{c} R\left(r\right)=\int_{-\infty }^{+\infty }U\left(k\right)H^{*}\left(k\right)e^{j2\pi k\cdot r}dk, \end{array}$$

where the superscript ∗ denotes complex conjugation. For pattern recognition purposes, (1) and (2) are equivalent ways to describe the process of correlation filtering defined in space domain and frequency domain, respectively.

It is clear from (1) and (2) that in general 3D correlation filtering requires 3D integration (in either the space or frequency domains). However, this is not the case when correlation filtering is applied to monochromatic optical fields propagating forward, typically the holographic reconstruction of optical fields by digital or optical means. In essence this is because *U*(**k**) is nonzero only within an area of a 2D surface, and consequently *u*(**r**) is highly correlated. 

According to scalar diffraction theory, the complex amplitude *u*(**r**) representing a monochromatic optical field propagation in a uniform dielectric must obey the Helmholtz equation [16] such that

(3) $$\begin{array}{c} \nabla^{2}u\left(r\right)+4\pi^{2}k^{2}u\left(r\right)=0, \end{array}$$

where *k* is a constant. Neglecting evanescent waves that occur close to boundaries and other obstructions, it is well known that the solutions to this equation are plane waves of the form:

(4) $$\begin{array}{c} u\left(r\right)=A\exp\left(j2\pi k\cdot r\right), \end{array}$$

where *A* is a complex constant. In these equations, *k* and **k** are the wave number and wave vector, respectively and are defined here such that *k* = |**k**| = 1/*λ*, where *λ* is wavelength. In consequence, any monochromatic optical field propagating a uniform dielectric is described completely by the superposition of plane waves such that

(5) $$\begin{array}{c} u\left(r\right)=\int_{-\infty }^{+\infty }U\left(k\right)\exp\left(j2\pi k\cdot r\right)dk, \end{array}$$

where *U*(**k**) is the spectral density, and *U*(**k**) is the Fourier transform of *u*(**r**) such that

(6) $$\begin{array}{c} U\left(k\right)=\int_{-\infty }^{+\infty }u\left(r\right)\exp\left(-j2\pi k\cdot r\right)dk. \end{array}$$

It is noted that because *u*(**r**) consists of plane waves of single wavelength, the values of *U*(**k**) only exist on an infinitely thin, spherical shell with a radius *k* = |**k**| = 1/*λ*. In consequence, if a general 3D correlation filter with transfer function *H*(**k**) is applied to a monochromatic optical field, *U*(**k**), then in frequency domain the product *U*(**k**)H*(**k**) is also nonzero only on the spherical shell and consequently will obey the Helmholtz equation. If we expand (5), we have

(7) u(rx,ry,rz)  =∭∞U(kx,ky,kz)exp⁡(j2π(kxrx+kyry+kzrz))δ      ×(kz±1λ2−kx2−ky2)dkxdkydkz  =∬∞U(kx,ky,±1λ2−kx2−ky2)     ×exp⁡(j2π(kxrx+kyry              ∓rz1λ2−kx2−ky2))dkxdky.

The square root in these equations represents light propagating through the *xy* plane in the positive and negative *z*-directions, respectively. Since most holographic recordings record the flux in only one direction, we will consider only the positive root. According to (7), we can define *U*_*z*_(*k*_*x*_, *k*_*y*_) as the 2D projection of the spectrum onto the plane, *k*_*z*_ = 0, such that

(8) $$\begin{array}{c} U_{z}\left(k_{x},k_{y}\right)=U\left(k_{x},k_{y},\sqrt{\frac{1}{\lambda^{2}}-k_{x}^{2}-k_{y}^{2}}\right). \end{array}$$

If *u*_*Z*_(*r*_*x*_, *r*_*y*_) represents the optical field in the plane *r*_*z*_ = *Z*, we have

(9) uZ(rx,ry)  =∬∞UZ(kx,ky)exp⁡(j2πZ1λ2−kx2−ky2)     ×exp⁡(j2π(kxrx+kyry))dkxdky.

In addition, taking the Fourier transform, we have

(10) UZ(kx,ky)  =exp⁡(−j2πZ1λ2−kx2−ky2)   ×∬∞uZ(rx,ry)exp⁡(−j2π(kxrx+kyry))drxdry.

Equation (10) allows the spectrum to be calculated from the knowledge of the optical field propagating through a single plane. Equation (9) allows the field in any parallel plane to be calculated. 

If we consider the application of a general 3D filter to the reconstruction of a propagating monochromatic field, we remember that the product *U*(**k**)H*(**k**) only exists on the surface of a sphere. Consequently, according to the derivation from (7) to (9), we have

(11) RZ(rx,ry)=∫−∞+∞UZ(kx,ky)Hz∗(kx,ky)    ×exp⁡(j2πZ1λ2−kx2−ky2)    ×exp⁡(j2π(rxkx+rxky))dkxdky,

where *R*_*Z*_(*r*_*x*_, *r*_*y*_) is the 3D correlation output in the plane *r*_*z*_ = *Z*, and

(12) $$\begin{array}{c} H_{Z}\left(k_{x},k_{y}\right)=H\left(k_{x},k_{y},\sqrt{\frac{1}{\lambda^{2}}-k_{x}^{2}-k_{y}^{2}}\right). \end{array}$$

Finally, we note that in the space domain the correlation is

(13) $$\begin{array}{c} R_{Z}\left(r_{x},r_{y}\right)=\int_{-\infty }^{+\infty }u_{Z}\left(u,v\right)h_{Z}\left(u-r_{x},v-r_{y}\right)du\;dv, \end{array}$$

where

(14) hZ(rx,ry)=∫−∞+∞HZ(kx,ky)    ×exp⁡(−j2π(rxkx+rxky))dkxdky.

Equation (13) shows that a single plane (*r*_*z*_ = *Z*) of the 3D correlation of a propagating optical field, *u*(**r**), with a general impulse response function, *h*(**r**), can be calculated as a 2D correlation of the field in that plane, *u*_*Z*_(*r*_*x*_, *r*_*y*_) with an impulse function, *h*_*Z*_(*r*_*x*_, *r*_*y*_), that is defined by (14). 

In the recent literature, 2D correlation filtering has been applied to complex images reconstructed from a digital holographic microscope [14]. Practically, a digital holographic microscope measures the complex amplitude in the plane of focus, and the complex amplitude images in the parallel planes are calculated based on optical propagation theory. It is noted that a linear filter that is designed to perform well in one plane of focus will not necessarily perform well in another, and therefore a nonlinear filtering process is required.

When the 3D complex amplitude distribution of samples is reconstructed from the digital holographic recording, correlation filters can be applied for pattern recognition. In the field of statistical pattern recognition, it is common to describe a digitized image of any dimension by the ordered variables in a vector [17], and we adopt this notation here. In this way, the discrete form of a complex 3D image can be written in vector notation by lexicographically scanning the 3D image array. Thus, an *n*-dimensional vector **x** = [*x*_1_, *x*_2_,…,*x*_*n*_]^*T*^ represents a 3D image with *n* volume elements. We define a correlation operator $\hat{H}$, with a filter kernel (or impulse response), **h** = [*h*_1_, *h*_2_,…,*h*_*n*_]^*T*^, is defined as,

(15) $$\begin{array}{c} \hat{H}x=\sum_{i=1}^{n}h_{i-n+1}^{*}x_{i}, \end{array}$$

where the superscript “∗” denotes the complex conjugate, and the subscript is taken to be modulo *n* such that

(16) $$\begin{array}{c} h_{n+a}=h_{a}. \end{array}$$

A nonlinear threshold operator $\hat{T}$ can be defined in the same way to operate on the individual components of a vector such that

(17) T^x=[ax13+bx12+cx1+d,ax23+bx22+cx2   +d,...,axn3+bxn2+cxn+d]T.

In general, image data from a hologram is a complex-amplitude field; however, we consider only the intensity distribution and define a modulus operator $\hat{M}$ that operates on the the output such that

(18) $$\begin{array}{c} \hat{M}x=\left[{\left|x_{1}\right|}^{2},{\left|x_{2}\right|}^{2},...,{\left|x_{n}\right|}^{2}\right]. \end{array}$$

In this way, a 3D nonlinear filter, $\hat{F}$, can be expressed as

(19) $$\begin{array}{c} \hat{F}=\hat{M}{\hat{T}}_{i}{\hat{H}}_{i}, \end{array}$$

where the subscript to each operator denotes the layer in which a given operator is applied.

Without loss of generality, we design the 3D nonlinear filter to generate a delta function for the objects to be recognized, and zero outputs for the patterns to be rejected. For this purpose, we define a matrix set, **S**, of *m* reference images such that **S** = [**s**_1_, **s**_2_,…, **s**_*m*_] and the corresponding output matrix, **R**, is given by

(20) $$\begin{array}{c} R=\hat{F}S. \end{array}$$

For the optimization of the 3D nonlinear filter, a matrix *O* with all the desired outputs intensity images is defined. In general, the desired outputs for in-class images will be a zero-valued vector with the first element set to be unit magnitude, and for an out-of-class image the desired output is zero. In order to train the filter with the desired performance, the error function below is requested to be minimized:

(21) $$\begin{array}{c} E=\sum_{i=1,\;j=1}^{n,m}{\left(R_{ij}-O_{ij}\right)}^{2}+n\sum_{j=1}^{m}{\left(R_{1j}-O_{1j}\right)}^{2}, \end{array}$$

where *R*_*ij*_ and *O*_*ij*_ represent the *i*th pixel of the *j*th training and output image, respectively. The first term in this expression is the variance of the actual output from the desired output. The second term represents the signal peaks (that for simplicity are defined to be the first term in the output vector) and is given extra weight to ensure that they have the desired unit magnitude. Because (21) is a nonlinear function with a large number of variables, it is not possible to find an analytical solution. Hence, an iterative method is used in the minimization process. In this case, a simulated annealing algorithm was implemented in the optimization because it is more likely to reach a global minimum [18].

In the practical implementations of the 3D nonlinear filter described in this paper, we require a filter to identify the presence of fairly small objects in a relatively large field. In these cases, a relatively small filter kernel is used, and the kernel is zero-padded to the same size as the input image. In the test of this paper, the training images are selected to be 32 × 32 × 16 elements, and we use 16 × 16 elements transfer function (2D). The filter output, the filter kernel, and the desired output images are all zero-padded to a resolution of 32 × 32 × 16 elements. In this way, edge effects in pattern recognition for large images can be avoided.

## 3. Experiment

The objective of the work described in this section was to demonstrate 3D rotationally invariance pattern recognition based on digital holographic microscopy for the classification of two species of live bacteria, *E. coli *and *Pantoea*. 

**Figure 1:**
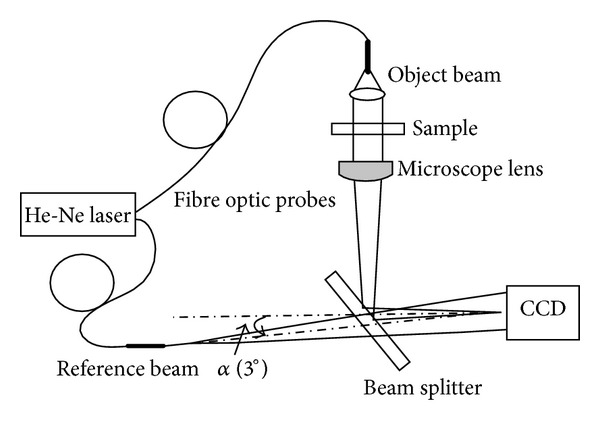
Holographic microscope with a coherent laser source.

The digital holographic microscope setup used for this study is illustrated in Figure 1. In this arrangement, a He-Ne laser (633 nm) is used as coherent light source and is divided by a beam splitter and launched into a pair of optical fibres of equal length. One fibre supplies the light that forms the object beam for the holographic recording and is collimated. The microscope is used in a transmission mode and has an objective lens with 100x magnification and an oil immersion objective with an equivalent numerical aperture of NA = 1.25. The object plane is imaged onto a CCD array placed approximately 200 mm from the objective. It is noted that because the microscope is holographic, the object of interest need not be located in the object plane. 

The fibre that supplies the reference beam has an open termination that is arranged to diverge from a point in the rear focal plane of the microscope objective. In this way, the interference of the light from the reference beam and the light scattered is recorded at the CCD. Phase curvature introduced by the imaging process [19] is precisely matched by the reference curvature, and straight interference fringes are observed in the image plane in the absence of any scattering objects. From the analysis in Section 2, we can see that the interference pattern recorded by the CCD can be demodulated to give the complex amplitude describing the propagating field in the object plane. For reasons of processing, efficiency care was taken to adjust the magnification of the microscope to match the CCD resolution such that an optimally sampled (Nyquist) reconstruction is produced.

**Figure 2:**
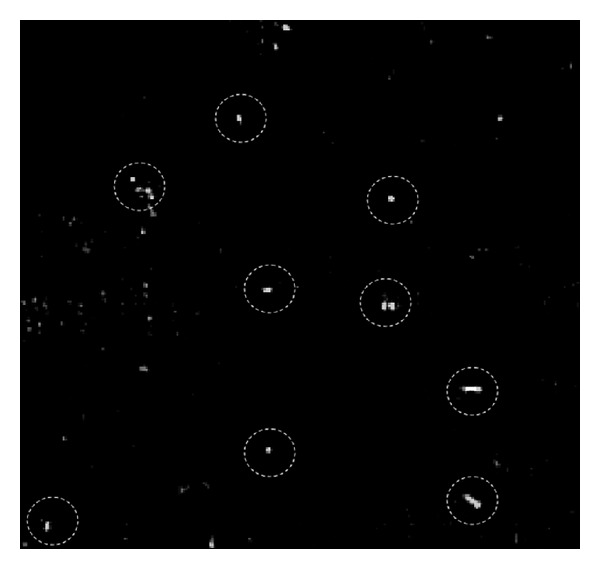
Holographic image with a field of view of 72 × 72 *µ*m (absolute value shown).

**Figure 3:**
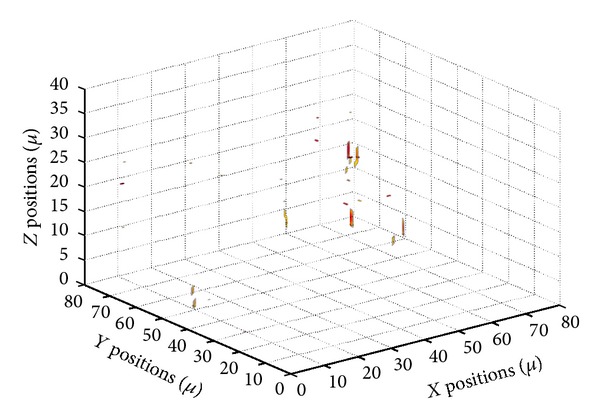
3D image of the optical field reconstructed from Figure 2.

The holographic microscope is implemented with a flow cell that defines an experimental volume. The nutrient fluid with two species of living bacteria, *E. coli *and *Pantoea*, is syringed into the flow cell through a pipe.  Figure 2 shows an image taken from the microscope corresponding to the absolute value of the complex amplitude in the object plane. In this image, the bacteria understood to be *E. coli *are highlighted with circles; some out-of-focus bacteria are invisible on this plane.  Figure 3 shows a 3D image of the field in Figure 2 reconstructed using the method demonstrated in the above section.

In this study, a 3D nonlinear filter was trained to highlight live *E. coli *bacteria floating in the flow cell, while the *Pantoea* bacteria will be ignored. However, the reference set preparation is one of the most challenging problems for the identification of the living cells because each of the live bacteria varies in size and shape and appears at random orientation. To recognise the bacteria regardless of their shapes and orientations, adequate representative distortions of bacteria images must be provided for the 3D nonlinear filter as reference images. 

**Figure 4:**
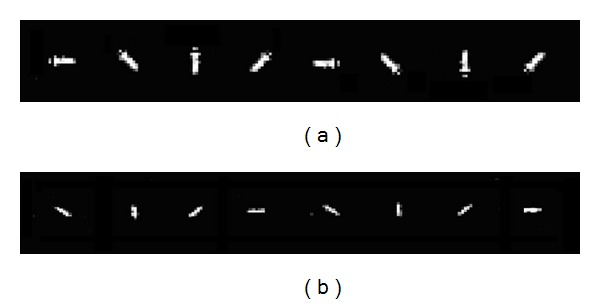
Typical bacteria (a) *E. coli *and (b) *Pantoea* in different rotated orientations.

The bacteria images registered as training set can be obtained by directly cropping the cell images from the 3D reconstructed field, or by simulating from the recorded images. For example, a selected bacteria image can be rotated to generate several orientation versions.  Figure 4(a) shows eight absolute value images of a typical rod-shaped *E. coli *rotated in steps of 45 degrees. *Pantoea* bacteria have a similar rod shape, but slightly different in size from *E*. *coli*.  Figure 4(b) shows one of the selected *Pantoea* in eight different rotated versions. 

**Figure 5:**
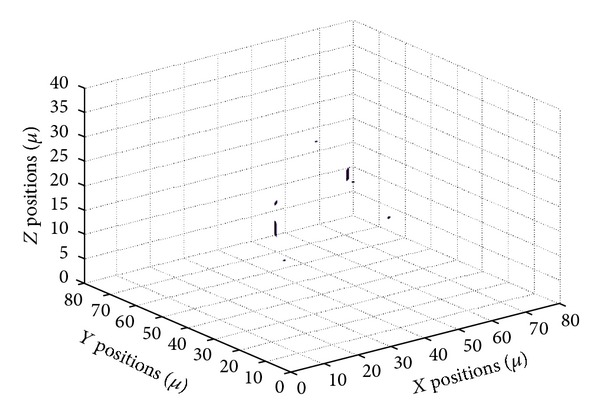
3D output for the 3D nonlinear filter trained to recognize *E. coli *(absolute amplitude value shown).

**Figure 6:**
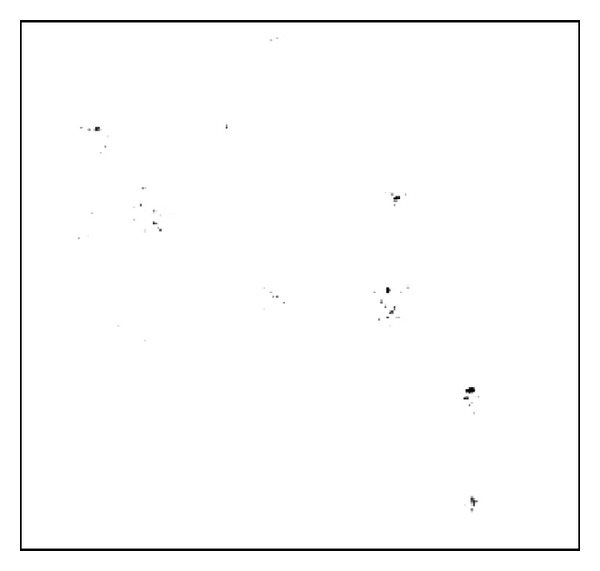
The projection of the output volume (absolute amplitude value shown).

To demonstrate the performance of the 3D nonlinear filter, we train the system to detect *E. coli *bacteria with 42 images, including 25 *E. coli *and 17 *Pantoea* images, and the filer is tested with the complex amplitude image in Figure 2.  Figure 5 shows the 3D image of the 3D filter output.  Figure 6 reports the projection of the output volume onto a plane. It can be seen that most of the *E. coli *bacteria had been highlighted by correlation peaks and the *Pantoea* had been ignored. However, a small portion of the *E. coli *cannot be detected; this is because the training set with limited number of reference images does not represent all the distortions and orientations of the bacteria. It is expected that classification rate can be improved if more reference images are included in the training set.

## 4. Conclusion

This paper describes 3D pattern recognition with a 3D nonlinear filter applied to monochromatic optical fields that can be recorded and reconstructed by holographic microscopes. The 3D extension and formulation of the nonlinear filter concept has been introduced. We have shown with experimental data that the 3D nonlinear filtering system provides additional capability as a means to perform 3D pattern recognition in a shift and rotationally invariant means. We demonstrate this in practice by applying the 3D nonlinear filter to a holographic recording of the light scattered from two kinds of living bacteria suspended in water. The experimental data demonstrated that the 3D nonlinear filter has good shift and rotationally invariant property in 3D space.

## References

[1] Minsky M (1988). Memoir on inventing the confocal scanning microscope. *Scanning*.

[2] Schnars U, Jüptner WPO (2002). Digital recording and numerical reconstruction of holograms. *Measurement Science and Technology*.

[3] Zhang T, Yamaguchi I (1998). Three-dimensional microscopy with phase-shifting digital holography. *Optics Letters*.

[4] Cuche E, Marquet P, Depeursinge C (1999). Simultaneous amplitude-contrast and quantitative phase-contrast microscopy by numerical reconstruction of Fresnel off-axis holograms. *Applied Optics*.

[5] Dubois F, Joannes L, Legros JC (1999). Improved three-dimensional imaging with a digital holography microscope with a source of partial spatial coherence. *Applied Optics*.

[6] Chen SY, Li YF, Guan Q, Xiao G (2006). Real-time three-dimensional surface measurement by color encoded light projection. *Applied Physics Letters*.

[7] Teng Z, Degnan AJ, Sadat U (2011). Characterization of healing following atherosclerotic carotid plaque rupture in acutely symptomatic patients: an exploratory study using in vivo cardiovascular magnetic resonance. *Journal of Cardiovascular Magnetic Resonance*.

[8] Lin L, Chen S, Shao Y, Gu Z (2013). Plane-based sampling for ray casting algorithm in sequential medical images. *Computational and Mathematical Methods in Medicine*.

[9] Guan Q, Du B (2012). Bayes clustering and structural support vector machines for segmentation of carotid artery plaques in multi-contrast MRI. *Computational and Mathematical Methods in Medicine*.

[10] Dubois F (1996). Nonlinear cascaded correlation processes to improve the performances of automatic spatial-frequency-selective filters in pattern recognition. *Applied Optics*.

[11] Reed S, Coupland J (2000). Statistical performance of cascaded linear shift-invariant processing. *Applied Optics*.

[12] Wu N, Alcock RD, Halliwell NA, Coupland JM (2005). Rotationally invariant pattern recognition by use of linear and nonlinear cascaded filters. *Applied Optics*.

[13] Javidi B, Tajahuerce E (2000). Three-dimensional object recognition by use of digital holography. *Optics Letters*.

[14] Dubois F, Minetti C, Monnom O, Yourassowsky C, Legros JC, Kischel P (2002). Pattern recognition with a digital holographic microscope working in partially coherent illumination. *Applied Optics*.

[15] Chen S, Zhao M (2012). Recent advances in morphological cell image analysis. *Computational and Mathematical Methods in Medicine*.

[16] Sommerfeld A (1949). *Partial Differential Equations in Physics*.

[17] Fukunaga K (1972). *Introduction to Statistical Pattern Recognition*.

[18] Kirkpatrick S, Gelatt CD, Vecchi MP (1983). Optimization by simulated annealing. *Science*.

[19] Goodman JW (1968). *Introduction to Fourier Optics*.

